# Aberrant methylation-mediated silencing of microRNAs contributes to HPV-induced anchorage independence

**DOI:** 10.18632/oncotarget.9698

**Published:** 2016-05-30

**Authors:** Saskia M. Wilting, Viktorian Miok, Annelieke Jaspers, Debby Boon, Hanne Sørgård, Malin Lando, Barbara C. Snoek, Wessel N. van Wieringen, Chris J.L.M. Meijer, Heidi Lyng, Peter J.F. Snijders, Renske D.M. Steenbergen

**Affiliations:** ^1^ Department of Pathology, VU University Medical Center, Amsterdam, The Netherlands; ^2^ Department of Epidemiology & Biostatistics, VU University Medical Center, Amsterdam, The Netherlands; ^3^ Department of Radiation Biology, Norwegian Radium Hospital, Oslo University Hospital, Oslo, Norway

**Keywords:** anoikis, cervical cancer, DNA methylation, CIN lesion

## Abstract

Cervical cancer and a subset of anogenital and head-and-neck carcinomas are caused by high-risk types of the human papillomavirus (hrHPV). During hrHPV-induced malignant transformation keratinocytes become able to grow anchorage independently, a tumorigenic trait at least partly associated with inactivation of tumor suppressor genes. We used hrHPV-containing keratinocytes to investigate the role of DNA methylation-mediated silencing of microRNAs (miRNAs) in the acquisition of anchorage independence.

Anchorage dependent (n=11) and independent passages (n=19) of 4 hrHPV-immortalized keratinocyte cell lines were treated with 2′-deoxy-5-azacytidine (DAC). Genome-wide miRNA expression profiles before and after treatment were compared to identify miRNAs silenced by methylation. Bisulfite sequencing and methylation-specific PCR showed increased methylation of hsa-mir-129-2/-137/-935/-3663/-3665 and -4281 in anchorage independent HPV-transformed keratinocytes and cervical cancer cell lines. Mature miRNAs derived from hsa-mir-129-2/-137/-3663 and -3665 showed functional relevance as they decreased anchorage independence in cervical cancer cell lines. Cervical (pre)cancerous lesions demonstrated increased methylation of hsa-mir-129-2/-935/-3663/-3665 and -4281, underlining the clinical relevance of our findings.

In conclusion, methylation-mediated silencing of tumor suppressive miRNAs contributes to acquisition of an anchorage independent phenotype. This study further substantiates the importance of miRNAs during early stages of carcinogenesis and underlines their potential as both disease markers and therapeutic targets.

## INTRODUCTION

Persistent infection with high-risk types of the human papillomavirus (hrHPV) is causally related to virtually all cervical cancers as well as a subset of other anogenital and head-and-neck carcinomas.

Normally, the viral life cycle of HPV is tightly linked to differentiation of the infected epithelium, resulting in very low expression of the viral oncogenes E6 and E7 in the basal dividing cells. In so-called transforming infections deregulated expression of E6 and E7 is found in the dividing cells. This aberrant expression results in uncontrolled cell proliferation and subsequent genetic instability. The latter is considered a driver of further cellular transformation [1]. Previous studies in which primary keratinocytes were transfected with either full length hrHPV types or with the viral oncogenes E6/E7 showed that hrHPV-induced transformation involves 4 distinct phenotypic stages: extended life span, immortality, anchorage independence and tumorigenicity [2, 3].

Anchorage independent growth is considered a crucial step in carcinogenesis and is often considered as surrogate marker for complete transformation *in vitro* [4, 5]. Epithelial cells are dependent on proper cell-cell and cell-matrix interactions (“anchors”) for both differentiation and proliferation. Loss of these interactions results in aberrant integrin signaling and subsequent induction of anoikis (detachment-induced cell death). Cancerous epithelial cells were shown to use various strategies to circumvent anoikis, including adaptation to the new environment by epithelial-to-mesenchymal (EMT) like de-differentiation or integrin switching, constitutive activation of survival and proliferation pathways, and temporary dormancy through either autophagy or entosis (reviewed by [6]). Cell fusion studies have shown that anchorage independence of hrHPV-transformed cells relies on a recessive event, suggesting that inactivation of tumor suppressor genes is involved in bypassing anoikis [2]. In support of this, functional loss of tumor suppressive host genes Caveolin-1, SOCS1, TAp63β, LKB1, CADM1, MAL, hsa-miR-34c-3p/5p and hsa-miR-203 was shown to affect anchorage independent growth of HPV-transformed cells [7–14].

Epigenetic changes represent an important mechanism for the silencing of tumor suppressor genes. In cervical cancer, DNA methylation-mediated silencing of a rapidly growing number of coding and non-coding genes is being found and functional relevance has been demonstrated for part of these genes [15–17]. Increased methylation has been shown for a number of miRNAs, including hsa-mir-34b, -95, -124, -125b1, -149, -203, -214, and -375 [13, 18–23]. However, so far a systematic investigation of the involvement of aberrantly methylated miRNAs in hrHPV-induced transformation is lacking.

In this study we used a well characterized *in vitro* model system consisting of 4 hrHPV-containing keratinocyte cell lines to thoroughly investigate the contribution of DNA methylation-mediated silencing of miRNAs to hrHPV-mediated transformation [24]. To this end genome-wide miRNA expression profiles were generated during different stages of transformation with and without demethylating 2′-deoxy-5-azacytidine (DAC) treatment. To select miRNAs (potentially) silenced by methylation, DAC-induced fold changes in mature miRNA expression were combined with DNA methylation levels measured by Infinium HumanMethylation450 BeadChip. Bisulfite sequencing and methylation-specific PCR (MSP) analysis were performed to verify DNA methylation both in cell lines and cervical (pre)cancerous tissue specimens. In addition, for all miRNAs that showed increased DNA methylation during HPV-induced transformation we determined effects of their ectopic expression on viability and anchorage independence of cervical cancer cell lines SiHa and CaSki.

## RESULTS

### Anchorage independence coincides with marked changes in overall miRNA expression

**Figure 1:**
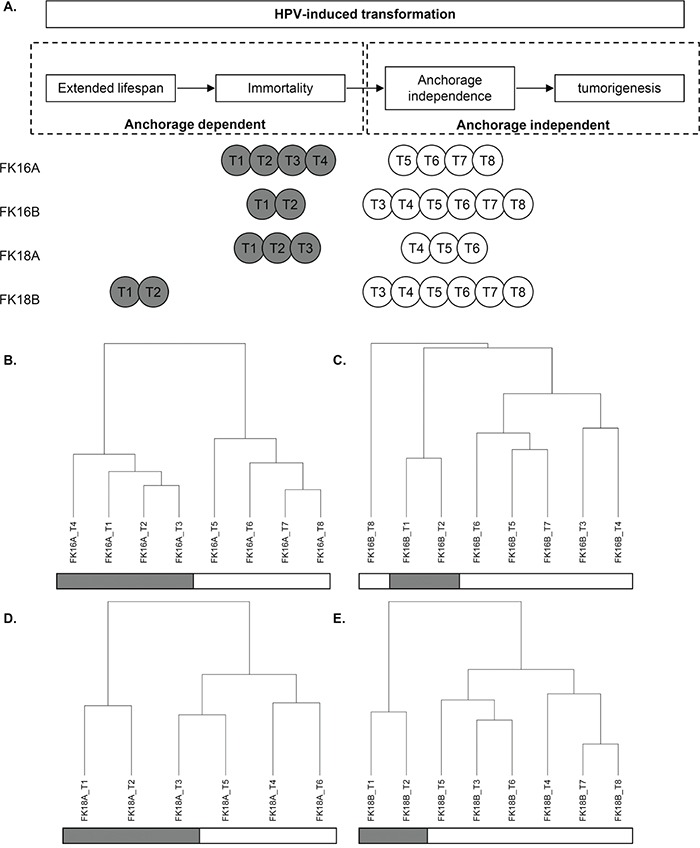
Characterization of our longitudinal *in vitro* model system of hrHPV-induced transformation In **A.** anchorage dependent (grey) and independent (white) timepoints (T) of all 4 cell lines are shown in relation to the transformation process. Unsupervised hierarchical cluster results based on overall miRNA expression are shown for **B.** FK16A, **C.** FK16B, **D.** FK18A, and **E.** FK18B. Anchorage dependent timepoints are marked in grey, whereas anchorage independent timepoints are white.

To study methylation-mediated silencing of miRNAs during HPV-induced transformation, we investigated 4 independent HPV-transformed keratinocyte cell lines at different stages during transformation. miRNA expression was determined in all 4 cell lines at 6-8 different passages (hereafter referred to as timepoints) with and without demethylating DAC treatment (Table 1). First we determined whether the selected timepoints represent distinct stages during HPV-induced transformation. hTERT expression, reflecting telomerase activation, was detectable at all timepoints for FK16A, FK16B and FK18A, suggesting that cells at all timepoints were already immortal (Figure 1A). In the first 2 timepoints of FK18B virtually no hTERT expression was detectable indicating these cells are still in their extended lifespan stage (Figure 1A). These findings are in concordance with previous telomerase activity assays done on these cell lines [24]. From all 4 cell lines both anchorage dependent and anchorage independent (i.e. fully transformed) timepoints were included (Table 1 and Figure 1A). Therefore our experimental set up allows further investigation of the contribution of methylation-mediated miRNA silencing to acquisition of anchorage independence.

**Table 1:** Passage numbers included for all 4 HPV-transformed cell lines

Timepoint	FK16A	FK16B	FK18A	FK18B
T1	p18	p21	p19	p17
T2	p22	p22	p21	p18
T3	p39	p45	p47	p40
T4	p52	p51	p99	p52
T5	p109	p89	p148	p90
T6	p115	p102	p160	p98
T7	p206	p140		p146
T8	p222	p169		p164

FK16A and FK16B contain HPV16, whereas FK18A and FK18B contain HPV18. Grey shaded passages represent anchorage dependent cells, whereas non-shaded passages are able to grow anchorage independently.

Interestingly, unsupervised clustering analysis of the baseline miRNA expression profiles (without DAC treatment) showed a clear distinction between anchorage dependent and anchorage independent timepoints in FK16A, FK16B and FK18B (Figure 1B, 1C and 1E). The highest anchorage dependent timepoint (T3) of FK18A clustered together with subsequent anchorage independent timepoints, suggesting that these FK18A T3 cells were on the verge of anchorage independence (Figure 1D). The stability of the clustering patterns was corroborated by consensus clustering [25]. These results indicate that changes in miRNA expression are associated with the acquisition of anchorage independence.

### Identification of miRNAs silenced by methylation during HPV-induced transformation

**Figure 2:**
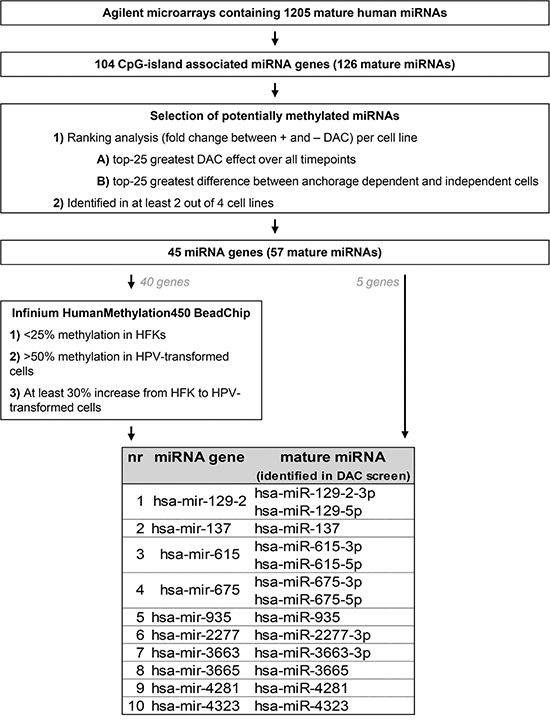
Schematic overview of the analysis pipeline used to select the most promising potential methylation targets

Next, we compared miRNA expression between DAC treated and untreated cells of all timepoints per cell line. Figure 2 summarizes the selection procedure we used to identify the most promising methylation candidates.

In total 104 CpG-island associated miRNA genes (as defined in Materials & Methods) encoding 126 mature miRNAs were included in the analysis. Per cell line we selected 1) the 25 miRNAs showing the highest upregulation upon DAC treatment over all timepoints and 2) the 25 miRNAs with the highest upregulation in anchorage independent timepoints. The resulting lists of candidate miRNAs were compared between the 4 cell lines and miRNAs were selected for further investigation if they were identified in 2 or more cell lines. This was the case for 57 mature miRNAs corresponding to 45 miRNA genes. An additional 16 mature miRNAs were only selected in 1 of the 4 cell lines and were therefore discarded from further analysis (Supplementary Table 1).

DNA methylation was measured for 40 of the 45 selected miRNA gene loci in primary keratinocytes (HFK) of 2 donors, FK16A T8, FK16B T7, FK18A T6 and FK18B T7 (all anchorage independent) using an Infinium HumanMethylation450 BeadChip. Genes showing 1) low methylation levels in primary keratinocytes (<25%), 2) at least 50% methylation in (part of) the HPV-transformed keratinocytes, and 3) a minimal increase in methylation of 30% from primary keratinocytes to HPV-transformed keratinocytes, were selected. In addition, we also selected the 5 miRNA genes for which no probes were available on the BeadChip. This resulted in selection of the following 10 miRNA genes for further investigation: hsa-mir-129-2, -137, -615, -675, -935, -2277, -3663, -3665, -4281, and -4323.

### Bisulfite sequencing analysis of the regulatory sequences of selected miRNA genes

**Figure 3:**
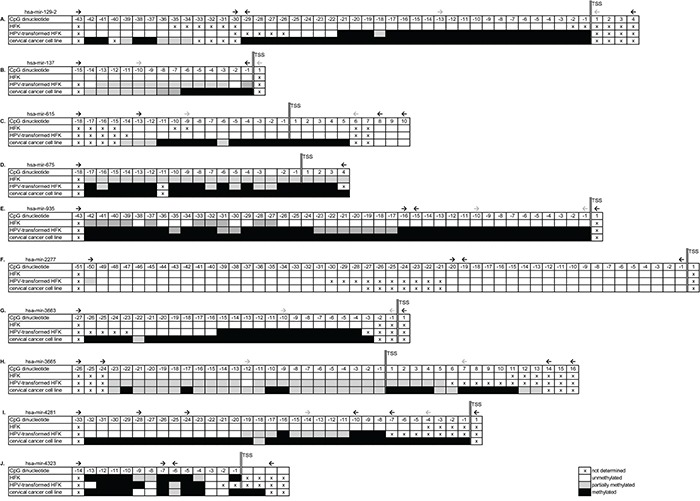
Bisulfite sequencing results of selected genomic regions in primary keratinocytes (HFK), HPV-transformed keratinocytes and cervical cancer cells Results are shown for **A.** hsa-mir-129-2, **B.** hsa-mir-137, **C.** hsa-mir-615, **D.** hsa-mir-675, **E.** hsa-mir-935, **F.** hsa-mir-2277, **G.** hsa-mir-3663, **H.** hsa-mir-3665, **I.** hsa-mir-4281, and **J.** hsa-mir-4323. Methylation-independent sequencing primers are indicated by black arrows and methylation-specific MSP primers are indicated by grey arrows. TSS; transcription start site.

Bisulfite sequencing was performed on HFKs, anchorage independent HPV-transformed keratinocytes (FK16A, FK16B, FK18A or FK18B), and cervical cancer cell lines (SiHa, HeLa or CaSki). For all 10 selected miRNA genes a region between 1000-500bp directly upstream of the transcription start site (TSS) was sequenced. Differential methylation in HPV-transformed keratinocytes and cancer cell lines compared to HFK was observed for hsa-mir-129-2, -137, -935 -3663, -3665, and -4281 (Figure 3A, 3B, 3E, 3G, 3H, and 3I, respectively). For hsa-mir-615 methylation was only observed in cervical cancer cells and not in HPV-transformed keratinocytes (Figure 3C), suggesting this to be a late event. No methylation was observed upstream of hsa-mir-2277 in any of the cells tested (Figure 3F). On the other hand, methylation upstream of hsa-mir-675 and -4323 was already observed in primary keratinocytes without HPV (Figure 3D and 3J). Based on these results, hsa-mir-675, -2277, and -4323 were excluded from further analyses.

### Aberrant hsa-mir-129-2, -137, -935, -3663, -3665, and -4281 methylation is common in HPV-transformed cells

**Figure 4:**
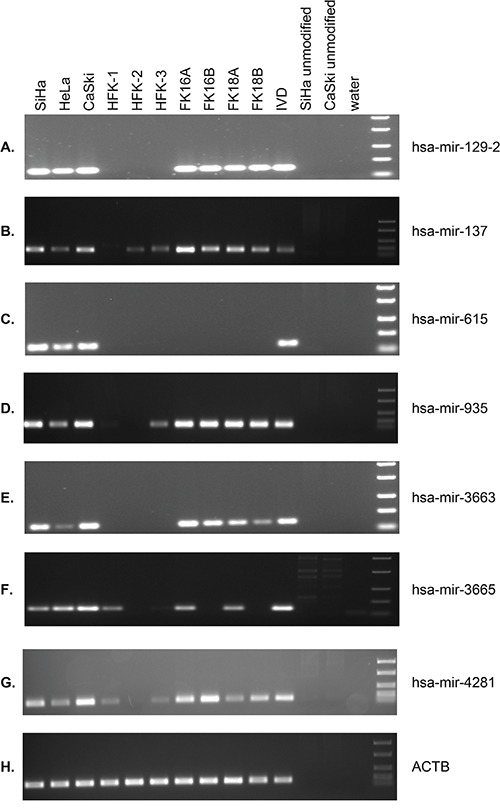
MSP results for selected miRNA genes in 3 cervical cancer cell lines (SiHa, HeLa, CaSki), primary keratinocytes (HFK) of 3 independent donors, and anchorage independent passages of 4 HPV-transformed keratinocyte cell lines (FK16A, FK16B, FK18A, and FK18B) Results are shown for **A.** hsa-mir-129-2, **B.** hsa-mir-137, **C.** hsa-mir-615, **D.** hsa-mir-935, **E.** hsa-mir-3663, **F.** hsa-mir-3665, **G.** hsa-mir-4281, and **H.** ACTB (reference gene). *In vitro* methylated DNA (IVD) and unmodified DNA were included as positive and negative controls, respectively.

To allow sensitive methylation analysis in larger numbers of samples, methylation-specific PCR (MSP) assays were designed for hsa-mir-129-2, -137, -615, -935, -3663, -3665, and -4281. Using the sequencing data described above we specifically designed primers to target the most differentially methylated region close to the TSS of the miRNA genes (indicated with grey arrows in Figure 3). MSP was performed on HFKs of 3 independent donors, anchorage independent passages of all 4 HPV-transformed keratinocyte cell lines (FK16A, FK16B, FK18A and FK18B) and the cervical cancer cell lines SiHa, HeLa, and CaSki (Figure 4). For hsa-mir-129-2 and -3663 no methylation was observed in any of the HFKs, whereas all HPV-transformed keratinocytes and cervical cancer cell lines showed clear methylation (Figure 4A and 4E). Concordant with sequencing results, methylation for hsa-mir-615 was only observed in the cervical cancer cell lines (Figure 4C and 3C). For hsa-mir-137, -935, -3665, and -4281 low levels of methylation were observed in part of the primary keratinocytes, but methylation clearly increased in most HPV-transformed keratinocytes and cervical cancer cell lines. The fact that with sequencing no methylation was observed for these miRNAs in HFK (Figure 3B, 3E, 3H and 3I) is probably due to the higher analytical sensitivity of MSP compared to sequencing [26, 27]. These results show that hsa-mir-129-2, -137, -935, -3663, -3665, and -4281 become hypermethylated during HPV-induced transformation. Methylation of hsa-mir-615, on the other hand, was only observed in cervical cancer cells and therefore does not seem to contribute to the process of transformation.

### miRNA silencing by DNA methylation contributes to anchorage independent growth

**Figure 5:**
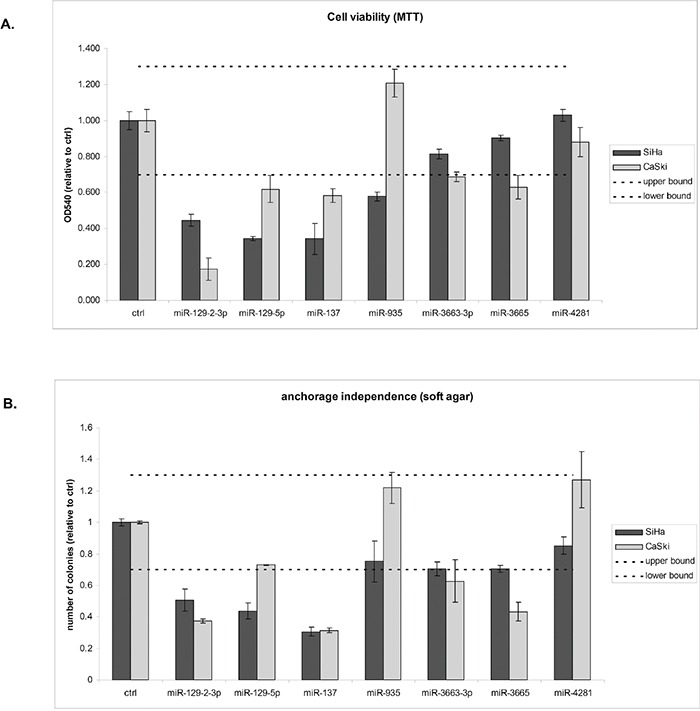
Functional effects of miRNAs silenced by methylation on HPV-induced transformation Effects of ectopic expression of mimics of all mature miRNAs derived from the methylated gene loci that showed a DAC effect (miR-129-5p, -129-2-3p, -137, -935, -;3663, -3665, and -4281) were determined on **A.** cell viability and **B.** anchorage independent growth. Dashed lines indicate the threshold of 30% we used to discriminate true effects from random variation. Results are representative of 2 independent experiments.

Next, we investigated whether silencing of the identified six hypermethylated miRNA genes contributes to the ability of cells to grow anchorage independently. For this purpose the corresponding mature miRNAs from the DAC screen were ectopically expressed in SiHa and CaSki cells, both of which were methylated for all six miRNA genes (Figure 3). Successful transfection of all mimics was verified by (quantitative) RT-PCR (data not shown). Subsequently effects on cell viability (Figure 5A) and anchorage independence were determined (Figure 5B). Based on the variation observed between replicate control experiments, only effects larger than 30% compared to the control transfected cells were considered true effects. miR-129-2-3p and -137 reduced both cell viability and anchorage independence in both cell lines tested. miR-4281 did not show any effect on viability and anchorage independence in either cell line. miR-129-5p reduced viability in both cell lines whereas anchorage independence was only reduced by this miRNA in SiHa. miR-3663-3p and -3665 modestly reduced viability and anchorage independence in CaSki but not in SiHa cells. However, a borderline reduction in anchorage independence was observed in SiHa cells as well. Finally, miR-935 modestly reduced viability in SiHa but not in CaSki cells. Together, these results support the functional relevance of methylation-mediated silencing of miRNAs for HPV-induced transformation.

### Increased methylation of hsa-mir-129-2, -935, -3663, -3665, and -4281 in cervical (pre)cancers

**Figure 6:**
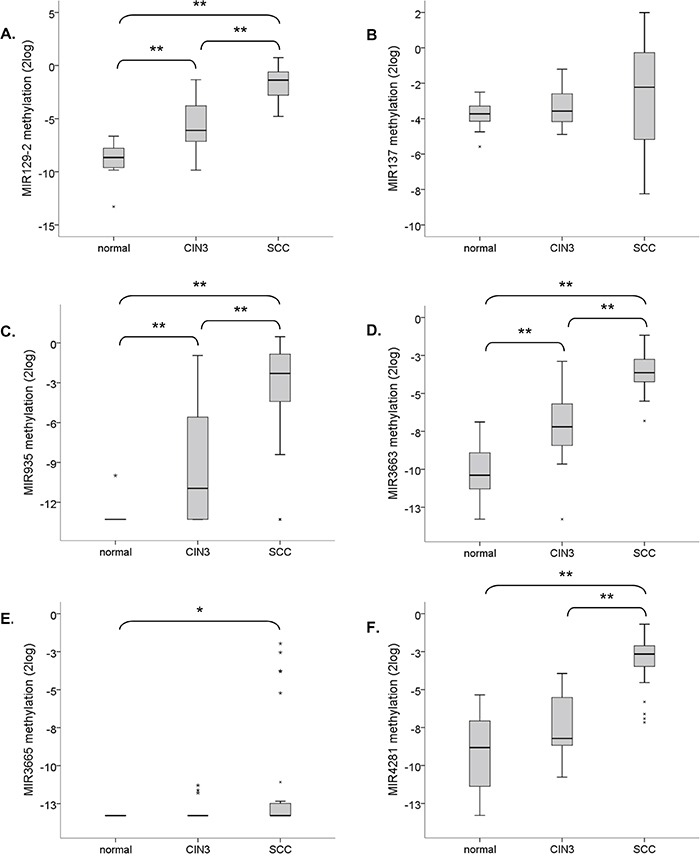
Methylation levels of selected miRNA genes relative to the reference gene (ACTB) in normal cervical tissue specimens (n=22), CIN3 lesions (n=23) and SCCs (n=28) Results are shown for **A.** hsa-mir-129-2, **B.** hsa-mir-137, **C.** hsa-mir-935, **D.** hsa-mir-3663, **E.** hsa-mir-3665, and **F.** hsa-mir-4281. ** indicates a two-sided p-value <0.001 (non-parametric Wilcoxon rank test); * indicates a two-sided p-value <0.05.

To verify the clinical relevance of our findings, methylation of hsa-mir-129-2, -137, -935, -3663, -3665 and -4281 was quantitatively determined in tissue specimens of normal cervical epithelium (n=22), CIN3 lesions (n=23), and SCCs (n=28). A significant increase in methylation levels with disease severity was observed for hsa-mir-129-2, -935, and -3663 (Figure 6A, 6C and 6D). For hsa-mir-4281 the increase in methylation from normal to CIN3 lesions was only borderline significant (p =0.059) but a significant increase was seen in SCCs compared to both normal and CIN3 samples (Figure 6F). Methylation of hsa-mir-3665 was absent in the large majority of samples but still showed a significant increase in SCCs (Figure 6E). On the other hand, hsa-mir-137 showed relatively high methylation levels in all samples with a small, non-significant, increase in SCCs (p=0.089).

In summary, methylation of hsa-mir-129-2, -935, -3663, -3665 and -4281 increases with disease severity in cervical tissue specimens.

## DISCUSSION

This study systematically investigated the contribution of methylation-mediated silencing of miRNAs to hrHPV-induced transformation over time. Using an unbiased longitudinal approach, we identified 6 miRNA genes showing progressive silencing by methylation during HPV-induced transformation (hsa-mir-129-2, -137, -935, -3663, -3665, and -4281). Mature miRNAs derived from hsa-mir-129-2, -137, -3663, and -3665 reduced cell viability and/or anchorage independent growth to various extents in SiHa and CaSki cervical cancer cells.

Unsupervised hierarchical clustering showed for the first time that anchorage dependent and anchorage independent timepoints within the same cell line could be discriminated based on their basal miRNA expression profiles. These clustering results suggest that alterations in miRNA expression are strongly associated with anchorage independent growth.

According to our definition only around 10% of the mature miRNAs measured were considered to come from CpG-island associated miRNA genes. This stringent definition limited the number of miRNAs included in the analysis. However, it was shown that in general DNA methylation in the region directly surrounding the TSS is most strongly associated with transcriptional silencing [28]. We were able to validate methylation for 2/3^rd^ of genes showing strong DAC-induced re-expression. The majority of these genes were already methylated in primary keratinocytes, a phenomenon observed by Lujambio *et al* as well [29].

Methylation of hsa-mir-129-2 has been described before in gastric, colorectal, renal, kidney, hepatocellular, esophageal, and endometrial carcinomas as well as in osteosarcomas [30–38]. Interferon-β dependent induction of hsa-miR-129-5p in cervical cancer cells was shown to reduce E6 and E7 expression via targeting of SP1 [39]. Additional targets described for hsa-miR-129-5p include SOX4, GALNT1, and VCP [33–35, 37, 40]. Hsa-miR-129-2-3p, which strongly reduced anchorage independence as well, was previously shown to reduce the levels of phosphorylated FAK protein and MMP2 and 9 proteins in renal carcinoma cells [41]. Interestingly, altered activation of the FAK signaling pathway has previously been described in HPV-immortalized keratinocytes and cervical cancer cell lines and was linked to invasion and anchorage independence [42–44].

Methylation of hsa-mir-137 has been described in oral and other head-and-neck, lung, bladder, breast, gastric, and colorectal cancers, glioblastomas, and neuroblastomas [31, 45–56]. Remarkably, in our hands hsa-miR-137 strongly reduced viability and anchorage independent growth but did not show increased methylation in (pre)cancerous tissue biopsies. Tumor suppressive effects were described for hsa-miR-137 before [45, 49] and in other cancer types cancer-specific methylation of mir-137 was observed as well. However, also in these studies, differences between normal and cancer were small (on average ~3x increase). Comparable to our results, also in these studies high background methylation was observed in normal samples [45, 53, 56]. Described targets of hsa-miR-137 so far include CDK6, LSD1, RTVP1, CAR and CDC42 [45, 46, 49, 54, 57, 58]. Increased expression of CDC42 is found in cervical (pre)cancers and was shown to promote formation of filopodia/pseudopodia thereby facilitating migration, a phenotype associated with anchorage independence as well [59, 60].

To the best of our knowledge, methylation of the other miRNA genes identified in this study has not been described before. Hsa-miR-935 was shown to be downregulated in medulloblastoma [61], whereas hsa-miR-3663-3p and -4281 conversely were found upregulated in malignant melanoma [62]. Hsa-miR-4281 and -935 did not influence anchorage independence, but they were specifically hypermethylated in (pre)cancers. Our results suggest that methylation of these miRNA genes is a relatively late event. Potentially, ectopic expression of these miRNAs therefore would have an influence on later phenotypes, such as invasion and tumorigenicity.

Mature miRNAs derived from 4 out of 6 identified silenced miRNA genes reduced anchorage independent growth. This fact underlines the value of longitudinal *in vitro* model systems for the identification of (epi)genetic events that contribute to (HPV-induced) transformation. In addition, significantly increased methylation levels of hsa-mir-129-2, -935, -3663, -3665, and -4281 were observed in cervical high-grade precancerous lesions (CIN3) and SCCs compared to normal cervical epithelium as well. This underscores the clinical relevance of our *in vitro* findings. The methylation status of these genes in low-grade cervical lesions (CIN1) and the heterogeneous group of CIN2 lesions, believed to encompass both low-grade and high-grade cervical precancerous disease [63], will be subject of future studies. In addition, these studies will address the potential marker value of the identified miRNA genes in cervical scrapes and self-collected cervico-vaginal specimens.

In summary, this study has shown that acquisition of anchorage independence during HPV-induced transformation *in vitro* is accompanied by clear changes in overall miRNA expression, which is partly attributable to aberrant DNA methylation. Mature miRNAs derived from genes silenced by methylation affected the ability of cervical cancer cells to grow anchorage independently, underlining the relevance of miRNA silencing for anchorage independent growth. Methylation levels of hsa-mir-129-2, -935, and -3663 were significantly higher in high-grade precancerous cervical lesions, suggesting these genes might represent promising biomarkers for the detection of precancerous lesions with a high immediate risk of cancer.

## MATERIALS AND METHODS

### Cell lines and clinical specimens

Establishment and culture of the HPV16 (FK16A and FK16B) and HPV18 (FK18A and FK18B) immortalized keratinocyte cell lines has been described previously [24]. Cervical carcinoma cell lines SiHa, HeLa and CaSki were obtained from the American Type Culture Collection (Manassas, VA, USA) and cultured as described previously [64]. Primary human foreskin keratinocytes (HFKs) were isolated and cultured as described previously [24]. From all 4 HPV-immortalized keratinocyte cell lines 6 to 8 different passage numbers (Table 1), including both anchorage dependent (grey shading in Table 1) and anchorage independent (no shading in Table 1) cells, were selected. To analyze the effect of methylation inhibition on the expression of miRNAs over time, cells were treated daily for 5 days with 5000 nM 5-aza-2′-deoxycytidine (DAC; Sigma-Aldrich, Zwijndrecht, The Netherlands) dissolved in PBS.

Formalin-fixed paraffin-embedded (FFPE) biopsies of 22 normal cervical squamous epithelial samples, 23 CIN3 lesions, and 28 SCCs were used. hrHPV testing of all biopsies was performed using the general primer GP5+/6(+)-mediated PCR-enzyme immunoassay method using a probe cocktail of 14 hrHPV types [65]. hrHPV was detected in 0% of normals, 96% of CIN3, and 100% of SCCs. The average age was 47.1 years (range 32-77) in the normal group, 34 years (range 26-45) in the CIN3 group and 48.2 years (range 30-85) in the SCC group. According to the International Federation of Gynecology and Obstetrics (FIGO) staging system all SCCs were stage I or II. All biopsies were collected during the course of routine clinical practice at the Department of Obstetrics and Gynecology at the VU University Medical Center (Amsterdam). This study followed the ethical guidelines of the Institutional Review Board of the VU University Medical Center.

### RNA/DNA isolation and DNA modification

Total RNA was isolated using TRIzol Reagent according to the manufacturer's instructions (Life Technologies, Carlsbad, CA, USA). RNA integrity was determined by gel electrophoresis. Total DNA was isolated from cell lines using the Purelink™ Genomic DNA Minikit (Life Technologies). DNA from FFPE biopsies was isolated by standard proteinase K digestion followed by phenol-chloroform purification [66].

Genomic DNA was modified using the EZ DNA Methylation kit (D5002, Zymo Research), which induces chemical conversion of unmethylated cytosines into uracils, whereas methylated cytosines are protected from this conversion.

### miRNA microarrays

Global miRNA expression profiles were determined in DAC treated and untreated cells using human miRNA microarrays (Sureprint G3 human v16 miRNA 8×60K; Agilent Technologies, Santa Clara, CA, USA) according to the manufacturer's instructions. These arrays contain 1205 human miRNAs based on the Sanger miRBase release 16. Microarray data are available from the NCBI Gene Expression Omnibus (GEO) through series accession number GSE78279 (http://www.ncbi.nlm.nih.gov/geo/query/acc.cgi?acc=GSE78279).

#### Data pre-processing

Probes corresponding to human miRNAs were selected and weakly correlating replicates of the same probe were removed. Data was normalized per treatment group using the robust quantile method and transformed using a variance stabilizing transformation [67, 68]. The obtained values were averaged per probe for further analysis.

#### Cluster analysis

Unsupervised hierarchical clustering was performed per cell line on complete miRNA expression profiles of untreated cells to explore the overall similarities in miRNA expression using maximum as distance measure and Ward's linkage.

#### Identification of potentially methylated miRNAs

To reduce identification of miRNAs indirectly affected by DAC treatment, only miRNAs with a CpG island close to the TSS were included for downstream analysis. For this we investigated whether (part of) the region ranging from 50 basepairs (bp) upstream to 25 bp downstream of the miRNA gene TSS was embedded in a CpG island according to the UCSC Genome Browser (www.genome.ucsc.edu) (GRCh37/hg19 assembly). Regions with a significantly higher density of CpG dinucleotides than found on average in the whole genome are considered CpG islands if 1) the GC content is at least 50%, 2) the region length is greater than 200 base pairs, and 3) the ratio of the observed number of CpG dinucleotides to the expected number based on the total number of Gs and Cs in the region is greater than 0.6 (ref [69]).

For all miRNAs having a CpG island in close genomic proximity, fold changes were calculated between DAC-treated and untreated cells per timepoint and cell line. Subsequently, miRNAs were ranked on this fold change in decreasing order and the top-25 miRNAs with the lowest median rank number over all passages were selected per cell line. In addition, we also selected the top-25 ranked miRNAs that showed the biggest difference between anchorage-dependent and anchorage-independent passages per cell line.

### Infinium HumanMethylation450 BeadChip analysis

Genome-wide methylation profiles were yielded from primary keratinocytes of 2 independent donors and FK16A T8, FK16B T7, FK18A T6 and FK18B T7 (see Table 1) by Infinium HumanMethylation450 BeadChip (Illumina, San Diego, CA, USA) according to the manufacturer's instructions. This platform interrogates 485 000 methylation sites, covering 99% of RefSeq genes and including miRNA promoter regions. Data are available from the NCBI Gene Expression Omnibus (GEO) through series accession number GSE78279 (http://www.ncbi.nlm.nih.gov/geo/query/acc.cgi?acc=GSE78279).

#### Data pre-processing

Data was processed and normalized using the pipeline described by Touleimat and Tost, which takes into account the different chemistry of class I and class II probes and returns Beta values representing the percentage of methylation for that particular CpG dinucleotide [70].

#### Selection of methylated miRNAs

Probes assigned to miRNA genes were selected and Beta values were compared between primary keratinocytes and HPV-transformed keratinocytes. Probes showing 1) low methylation levels in primary keratinocytes (<25%), 2) at least 50% methylation in (part of) the HPV-transformed keratinocytes, and 3) a minimal increase in methylation of 30% from primary keratinocytes to (part of) HPV-transformed keratinocytes were selected.

### Bisulfite sequencing

Bisulfite sequencing was performed on a 500-1000bp region directly upstream of the TSS in primary keratinocytes, HPV-transformed keratinocytes and cervical cancer cell lines SiHa, HeLa and/or CaSki. Sequencing primers are listed in Supplementary Table S2A and primer locations are indicated in Figure 3. Sequencing was performed on an AB 3500 Genetic Analyzer (Life Technologies) using the Big dye terminator v3.1 sequencing kit (Life Technologies) according to the manufacturer's instructions.

### (Quantitative) methylation-specific PCR ((q)MSP) analysis

MSP analysis was performed as described previously for selected miRNAs [10]. Primers were designed to amplify the methylated DNA sequence of the selected genomic region (Supplementary Table S2B and Figure 3). In addition, the modified sequence of the housekeeping gene β-actin (ACTB) was amplified to verify sufficient DNA quality and successful DNA modification [71]. *In vitro* methylated DNA (IVD, Qiagen, Venlo, The Netherlands) and unmodified DNA were included as positive and negative control respectively.

For hsa-mir-129-2, -137, -935, -3663, -3665, and -4281 a methylation specific probe was used for quantitative measurement of methylation levels as described previously (Supplementary Table S2B) [21]. Assays were run in a multiplex format as described previously on the ABI 7500 Fast Real-Time PCR System (Life Technologies) [72]. Methylation values were normalized to ACTB using the comparative CT method (2^−ΔCT^) [73].

### Quantitative reverse transcription-PCR (qRT-PCR)

Expression of hsa-miR-129-2-3p (assay ID: 001184), hsa-miR-129-5p (assay ID: 00590), hsa-miR-137 (assay ID: 001129), hsa-miR-3663-3p (assay ID: 465775), hsa-miR-935 (assay ID: 002178), and the small nucleolar RNA transcript RNU43 (assay ID: 001095) was measured using TaqMan microRNA assays following the manufacturer's instructions (Life Technologies) on the ABI 7500 Fast Real-Time PCR System (Life Technologies). miRNA expression values were normalized to RNU43 using the comparative CT method (2^−ΔCT^) [73].

For hsa-miR-3665 and hsa-miR-4281 we designed primers following the guidelines described previously (Supplementary Table S2C) [74]. Resulting PCR products were cloned using the TOPO TA cloning kit (Life Technologies) and sequenced to verify the specificity of the designed primers (data not shown). hTERT expression was determined using primers and probe described by Buttitta et al [75]. Expression levels were normalized to snRNP U1A levels as described before [76].

### Transfection with miRNA mimics

SiHa and CaSki cells were transiently transfected with 30nM of miRIDIAN microRNA mimics for hsa-miR-129-2-3p (C-301063-01), hsa-miR-129-5p (C-300539-03), hsa-miR-137 (C-300604-07), hsa-miR-3663-3p (C-301543-00), hsa-miR-3665 (C-301545-00), hsa-miR-4281 (C-3018242-00), hsa-miR-935 (C-301264-01) or negative control #2 (CN-00200-01; GE Healthcare Dharmacon Inc., Lafayette, CO 80026, USA) according to the manufacturer's instructions. Cells were transfected for 18 hours using Dharmafect 2 (GE Healthcare Dharmacon Inc.).

### Cell viability assay

Cell viability was measured using a colorimetric (MTT-tetrazolium) assay (MP Biomedicals, Santa Ana, CA, USA) as described before [10]. In this assay the amount of dye conversion, as measured by the optical density at a wavelength of 540 nm, is directly related to the number of viable cells in each well. Cells were seeded in triplicate 24 hours after transfection in 96 well plates (2500 cells/well) and viability was measured at day 0 and at day 5. The average measurement of day 0 was subsequently subtracted from the measurement at day 5. Two independent experiments were performed. Based on the variation observed between control transfected cells in separate experiments (~10%), cell viability was considered to be altered if it deviated by 30% or more.

### Anchorage independent growth

Colony formation in soft agar was analyzed as described before [12]. In short, 24 hours after transfection 5000 cells were suspended in medium containing 0.35% top agarose (Seaplague agarose; Lonza Group Ltd., Basel, Switzerland) and plated in duplicate on a surface of 0.6% bottom agarose in 6-cm dishes. Cells were incubated at 37°C for 3 weeks and were fed weekly by overlaying the agarose with fresh medium. After 3 weeks colonies were photographed and counted. Two independent experiments were performed. Based on the variation observed between control transfected cells in separate experiments (~10%), anchorage independent growth was considered to be altered if it deviated by 30% or more.

### Statistical analysis

Differences in methylation levels between clinical sample groups were determined using the non-parametric Wilcoxon rank test. A two-sided p value below 0.05 was considered statistically significant.

## SUPPLEMENTARY TABLE




