# Supplementary material for: Aberrant methylation-mediated silencing of microRNAs contributes to HPV-induced anchorage independence
Source: Oncotarget. 2016 May 30;7(28):43805–19. doi: 10.18632/oncotarget.9698 (PMC5190061; doi:10.18632/oncotarget.9698)
Supplement: Supplementary file 1 [file oncotarget-07-43805-s001.pdf]

Aberrant methylation-mediated silencing of microRNAs contributes to HPV-induced anchorage independence

SUPPLEMENTARY TABLE

Supplementary Table S1: miRNA genes potentially silenced by methylation that were only identified in 1 out of 4 cell lines investigated

nr	miRNA gene (CpG island associated)	cell line	mature miRNA
1	hsa-mir-1237	FK16A	hsa-miR-1237
2	hsa-miR-1292	FK16A	hsa-miR-1292
3	hsa-mir-1538	FK16B	hsa-miR-1538
4	hsa-mir-2861	FK16B	hsa-miR-2861
5	hsa-mir-3178	FK16A	hsa-miR-3178
6	hsa-mir-3180-1/-3	FK16A	hsa-miR-3180-5p
7	hsa-mir-3613	FK18A	hsa-miR-3613-3p
8	hsa-mir-3615	FK16B	hsa-miR-3615
9	hsa-mir-3652	FK16A	hsa-miR-3652
10	hsa-mir-564	FK18B	hsa-miR-564
11	hsa-mir-572	FK16B	hsa-miR-572
12	hsa-mir-632	FK18A	hsa-miR-632
13	hsa-mir-638	FK16B	hsa-miR-638
14	hsa-mir-1908	FK16B	hsa-miR-1908
15	hsa-mir-611	FK16A	hsa-miR-611
16	hsa-miR-943	FK16B	hsa-miR-943

SupplementaryTable S2: Sequences for A. bisulfite sequencing primers, B. (q)MSP primers and probes, C. RT-PCR primers

See Supplementary File 1
