# Supplementary material for: Aberrant methylation-mediated silencing of microRNAs contributes to HPV-induced anchorage independence
Source: Oncotarget. 2016 May 30;7(28):43805–19. doi: 10.18632/oncotarget.9698 (PMC5190061; doi:10.18632/oncotarget.9698)
Supplement: Supplementary file 2 [file oncotarget-07-43805-s002.doc]

**Table 2.** Sequences for **A.** bisulfite sequencing primers, **B.** (q)MSP primers and probes, **C.** RT-PCR primers.

| **A.** | **Gene** | **oligo** | **sequence** | **Tm (°C)** | **size (bp)** |
| --- | --- | --- | --- | --- | --- |
|  | mir-129-2 | Forward | GAGGGAGGAGTTTTATTTAGTTG | 52.3 | 318 |
|  |  | Reverse | TCACCCACCAATCAAAAAA | 53.4 |  |
|  |  | Forward | TAATATGTTTTAGTTTAGGTTTTGTTG | 52 | 345 |
|  |  | Reverse | ACCTATCCTATCCCTCTATCTCC | 52.5 |  |
|  |  | Forward | GGAGATAGAGGGATAGGATAGGT |  | 474 |
|  |  | Reverse | ACTATTAAATTATATACAACAAACCCAAAC | 54.5 |  |
|  | mir-137 | Forward | GTTATTTGGATTTGGGTAGGAAGTAG | 56.7 | 226 |
|  |  | Reverse | AAAAAAATCAAAAAACCAAACTACC | 55.3 |  |
|  | mir-615 | Forward | TGTTTATTAAAAGGTTTAGAGGTTGTGT | 56.3 | 336 |
|  |  | Reverse | TAAAAAAAAAAAAAAAACCCAAACTC | 56.6 |  |
|  |  | Forward | AGAGGGATTTGAAGGGTGAG | 54.4 | 271 |
|  |  | Reverse | CCCAAACAAAAAACTTTCCAC | 55 |  |
|  | mir-675 | Forward | TAATTTATTTAGTAGTAGGTATAGGGGTAGT | 52.8 | 334 |
|  |  | Reverse | AACTCCCCTAAATACTATACTATCTACC | 52.1 |  |
|  | mir-935 | Forward | TTGGGTTAGTTAGATTTAGG | 45.5 | 260 |
|  |  | Reverse | AATAATAATTTTTCTTCTCCTC | 45.4 |  |
|  |  | Forward | GAGGAGAAGAAAAATTATTATT | 45.4 | 158 |
|  |  | Reverse | CCAACCTTAAACAAATCC | 46.5 |  |
|  | mir-2277 | Forward | TTGAGTTAGGGGGTAAGTGAT | 51.8 | 344 |
|  |  | Reverse | ACTTCAAAAAACTAATCTTAACCTCTA | 52.2 |  |
|  |  | Forward | GAGTTAATTTTTTGGAAGAGTTTTT | 52.9 | 317 |
|  |  | Reverse | CCCATATTACTTTAACAATTACACTC | 51.8 |  |
|  | mir-3663 | Forward | TGTAAGGTTTAGTATTTAGTGGTTATTTATTA | 54.1 | 375 |
|  |  | Reverse | AAAACCCTCCTCCCCTTC | 54.1 |  |
|  | mir-3665 | Forward | GGGGTTAGGTAGGTAGAAGGTGT | 55.3 | 282 |
|  |  | Reverse | (G)AAACTAAAAAAACAAAAC(G/A)AACAACTA | 55.3/53.6 |  |
|  | mir-4281 | Forward | TTTAGGTTTTAAGGACGTTAGGA | 53.3 | 381 |
|  |  | Reverse | AAAAACCCCCAACCCTAC | 52.5 |  |
|  |  | Forward | GGGTTTAGGGTTTAGAAGGG | 53.3 | 210 |
|  |  | Reverse | ATAACCCCCTACTTAATTTTCTC | 51.1 |  |
|  |  | Forward | GGGGTTGTAGTTTGTAGGGTT | 53.3 | 308 |
|  |  | Reverse | CCCTCCTTAACCATAAACAAAC | 53.3 |  |
|  | mir-4323 | Forward | GGATTTTTTTTGTGAAATGGTT | 53.8 | 328 |
|  |  | Reverse | TCCCATATACTAATACCTCCTCTACTC | 53.9 |  |
|  |  | Forward | GTTTTTGTGTTTATGGGTTTATGT | 53.2 | 288 |
|  |  | Reverse | CCAAACACCCCACATACC | 52.8 |  |
|  |  |  |  |  |  |
| **B.** | **Gene** | **oligo** | **sequence** | **Tm (°C)** | **size (bp)** |
|  | mir-129-2 | Forward | GCGGAGTGGTGAGATTGAGTC | 58.3 | 120 |
|  |  | Reverse | AAAATATACCGACTTCTTCGATTCG | 58 |  |
|  |  | Probe | DFO-CCTAAAACCGAACAAACTAAATCTCCCCAACG-BHQ2 | 69.2 |  |
|  | mir-137 | Forward | CGGGTTTAGCGAGTAGTAAGAGTTTTG | 60.7 | 115 |
|  |  | Reverse | GAAAAAAATCAAAAAACCAAACTACCG | 60.3 |  |
|  |  | Probe | JOE- GCTACTACTACCGCCGCCGCCG -BHQ1 | 69.3 |  |
|  | mir-615 | Forward | TTTTTTGCGGAGTCGGTTTC | 58.8 | 108 |
|  |  | Reverse | CAATAAACACCCTCGAAATCCG | 59.3 |  |
|  | mir-935 | Forward | GAGGTGATAGGCGTGTTGGTC | 58.1 | 88 |
|  |  | Reverse | CAACCTTAAACAAATCCGAACG | 57.4 |  |
|  |  | Probe | DFO- GCCTCGCGACTACGCTCGATATAAATATTAAC -BHQ2 | 66.6 |  |
|  | mir-3663 | Forward | GCGGGGAGGGGTTGTTC | 59.8 | 110 |
|  |  | Reverse | AAAAAAACCAATTAAAAAATCACAATCG | 59.8 |  |
|  |  | Probe | FAM-CGAAAAAACAATAAAAAACGAAAAACACGAAACGA-BHQ1 |  |  |
|  | mir-3665 | Forward | GAGTTATCGTCGTTGTTATTATCGTTGTC | 60.3 | 107 |
|  |  | Reverse | CCCCGACCGCCACG | 60.1 |  |
|  |  | Probe | JOE- CGACCTCAAAAAACCTAAAACTCGAACTAACGCT -BHQ1 | 69.1 |  |
|  | mir-4281 | Forward | GTTTTTTTTAGGTCGTTAGGATGGAC | 58.6 | 115 |
|  |  | Reverse | TTCTCCGCCGCCTCG | 59.1 |  |
|  |  | Probe | 5’-FAM-ATAACCCCCTACTTAATTTTCTCCGCGACTACC-BHQ1-3' | 68.1 |  |
|  |  |  |  |  |  |
| **C.** | **Gene** | **oligo** | **sequence** | **Tm (°C)** | **size (bp)** |
|  | miR-3665 | looped RT | GTCGTATCCAGTGCAGGGTCCGAGGTATTCGCACTGGATACGACCGCCGC |  |  |
|  |  | Forward | GTGAGCAAGCAGGTGCGG | 60.1 |  |
|  |  | Reverse | GTGCAGGGTCCGAGGT | 53.4 |  |
|  | miR-4281 | looped RT | GTCGTATCCAGTGCAGGGTCCGAGGTATTCGCACTGGATACGACCCCCCC | 59 |  |
|  |  | Forward | TAGCTAGGGTCCCGGGGA | 53.4 |  |
|  |  | Reverse | GTGCAGGGTCCGAGGT |  |  |

Size (bp) refers to the amplicon size in basepairs.
